# Using Sit-Stand Workstations to Decrease Sedentary Time in Office Workers: A Randomized Crossover Trial

**DOI:** 10.3390/ijerph110706653

**Published:** 2014-06-25

**Authors:** Nirjhar Dutta, Gabriel A. Koepp, Steven D. Stovitz, James A. Levine, Mark A. Pereira

**Affiliations:** 1 Division of Health Policy & Management, School of Public Health, University of Minnesota, Minneapolis, MN 55455, USA; E-Mail: dutta.nirjhar@gmail.com; 2 Endocrine Research Unit, Mayo Clinic, Rochester, MN 55905, USA; E-Mails: koepp.gabriel@mayo.edu (G.A.K.); levine.james@mayo.edu (J.A.L.); 3 Family Medicine and Community Health, Medical School, University of Minnesota, Minneapolis, MN 55455, USA; E-Mail: stovitz@umn.edu; 4 Division of Epidemiology & Community Health, School of Public Health, University of Minnesota, Minneapolis, MN 55454, USA

**Keywords:** sedentary time, sit stand desk, work place intervention, accelerometer, dietary assessment

## Abstract

*Objective*: This study was conducted to determine whether installation of sit-stand desks (SSDs) could lead to decreased sitting time during the workday among sedentary office workers. *Methods*: A randomized cross-over trial was conducted from January to April, 2012 at a business in Minneapolis. 28 (nine men, 26 full-time) sedentary office workers took part in a 4 week intervention period which included the use of SSDs to gradually replace 50% of sitting time with standing during the workday. Physical activity was the primary outcome. Mood, energy level, fatigue, appetite, dietary intake, and productivity were explored as secondary outcomes. *Results*: The intervention reduced sitting time at work by 21% (95% CI 18%–25%) and sedentary time by 4.8 min/work-hr (95% CI 4.1–5.4 min/work-hr). For a 40 h work-week, this translates into replacement of 8 h of sitting time with standing and sedentary time being reduced by 3.2 h. Activity level during non-work hours did not change. The intervention also increased overall sense of well-being, energy, decreased fatigue, had no impact on productivity, and reduced appetite and dietary intake. The workstations were popular with the participants. *Conclusion*: The SSD intervention was successful in increasing work-time activity level, without changing activity level during non-work hours.

## 1. Introduction

Physical activity (PA) guidelines call for 2.5 h of moderate intensity aerobic exercise and also some muscle strengthening training per week—In total, about 3.5 h per week of purposeful exercise [1]. Given that the average person sleeps 8.5 h/day, there are 15.5 waking h/day, or 108.5 total waking h/week [2]. This leaves, even for a person who meets exercise guidelines, 105 h/week when one is not purposefully exercising or sleeping. PA during this time falls under sedentary (*i.e.*, standing in line, sitting, or lying down; 1–1.5 MET) and light activity (*i.e.*, moving about, or leisurely walking at < 3 mph; 1.6–2.9 MET) [3]. Decreasing sedentary time and increasing light activity during the waking hours may be a critical component of body weight regulation and chronic disease risk [4].

People who spend most of their waking hours in sedentary time are at higher risk for adverse health outcomes, even if they exercise the same amount as those with less sedentary lifestyle [5,6]. Breaks in sedentary behavior have beneficial health effects in terms of cardio-metabolic risk factors [7,8,9]. Given that working adults in developed countries typically spend at least half of their working day sitting, the workplace is an appropriate site for interventions aimed at reducing sedentary time [10,11]. Only very recently have experimental studies attempted to replace sitting time with standing in natural environments of the workplace and classroom [12,13,14,15].

The goal of this study was to examine the effects of using an adjustable sit-stand desk (SSD) in the workplace in terms of sitting, standing, and light activity. Specifically, it was hypothesized that the amount of sitting time would be lower and light activity would be higher during work hours when the employees were using SSDs compared to their usual sitting desks. Effects on perceived energy, fatigue, appetite, productivity, and dietary intake were examined as secondary outcomes.

## 2. Experimental Section

The study was approved by the University of Minnesota’s institutional review board and was registered on clinicaltrials.gov (NCT01863056). Written informed consent was obtained from all participants. 

### 2.1. Study Design

A randomized cross-over study of office workers at Caldrea, Inc., a company located in the Twin Cities Metro Area, MN, USA, was conducted from January to April 2012. The cross-over design was chosen as the most efficient scientific approach to test the feasibility and potential effects of using SSDs in the context of a relatively modest, short-term study. The office consisted of one floor of a building (2581 m^2^) and included approximately 50 employees. The cross-over design included a 4-week sit-stand intervention period and a 4-week control period, separated by a 2-week washout/usual habits period. The first phase of the study took place during January–February, the washout in February–March, and the second phase during March–April. 

### 2.2. Eligibility

Eligible participants were adult (aged 18 years and over) employees of the company who were sedentary during the majority of the workday and used a single computer workstation for at least 20 h per week. Participants had to be willing to stand for 50% of the workday because this was the behavioral target for the intervention. Exclusion criteria included contraindications to standing at work, such as musculoskeletal problems, autoimmune conditions, varicose veins, and pregnancy; nobody met these criteria.

### 2.3. Recruitment

A word-of-mouth search was performed for finding interested companies to host the study and *Caldrea Inc*. volunteered. A recruitment presentation was made at an all-employee meeting (n ~ 50) and was followed, a few days later, by enrollment interviews that covered inclusion/exclusion criteria, demographic information, and work schedule. 

### 2.4. Intervention

Based on randomization, either the first or third month involved an active intervention to use an adjustable SSD with the goal of gradually replacing 50% of sitting time over the month with standing time at work. This study goal was arrived at through discussions with ergonomic experts and researchers in the field. The desks were provided and installed by Ergotron, Inc. (Eagan, MN, USA). Three different models of desks were used to best match the need of the participants: Workfit-S^®^, a setup that attaches to the front of one’s existing desk that can hold computer monitor, keyboard, and mouse; Workfit-A^®^, a setup that is identical to Workfit-S^®^ but attaches to the back of one’s existing desk; Workfit-D^®^, a whole desk that is easily moved up and down. The Workfit-A and S^®^ also came with an added work-surface and all three types of desks came with anti-fatigue floor mats for comfort during standing. Users switched from sitting to standing by pushing on a lever on the front of the desk. An ergonomic evaluation were provided to each participant on proper standing/sitting height for the workstation. An email was sent at the beginning of each week reminding participants of the study goal of replacing 50% of their sitting time at work with standing. 

### 2.5. Control

During the control period, participants were asked to maintain their usual work habits, which primarily involved working from their company provided desks and chairs (all participants had identical desks and chairs). During the control period, all the same measurements were made as during the intervention period. They also received an ergonomic evaluation. 

### 2.6. Wash-out

The washout was essentially identical to the control period, except no measurements were taken and no contact was made with the participants. 

### 2.7. Primary Outcome

The primary outcomes for the study were sitting time, standing time, and light activity at work. Sitting and standing time were objectively assessed on two random days each week by an accelerometer (Modular Signal Recorder 145, MSR Electronics GmbH, Seuzach, Switzerland) which participants wore on their lower thigh (unpublished data from MSR validation study: Compared with the validated PA Monitoring System (PAMS), the MSR distinguished sedentary activity in various body postures and walking activity; N = 7, intra-class correlation coefficient (*r*^2^ > 0.95)). Similar thigh mounted device have been used in previous studies to monitor activity [16,17]. 

Sitting and standing time were also measured subjectively via the self-reported Occupational Sitting and PA Questionnaire (OSPAQ) [18]. The survey was loaded onto a study-specific survey website hosted by SurveyMonkey.com (Palo Alto, CA, USA) and a link to the survey was emailed to the participants at the end of each week. Percent of time spent sitting, standing, walking, and heavy activity for each week was determined. Time spent at work was tracked via another online survey asking about when an employee was physically present at the worksite each week. 

PA was measured with a validated accelerometer (Gruve*^®^*, Muve Inc. Minneapolis, MN, USA), which participants wore on the hip during all waking hours [19]. Raw data from the accelerometer were analyzed in activity units per hour (AU/hr). The AU/hr was then converted to the four types of activity: sedentary, light, moderate, and intense (by proprietary algorithms of Gruve using participant’s age, sex, height, and weight, which was collected at baseline using calibrated scales). Sedentary activity is defined as 0 to 1.6 metabolic equivalent of task (MET), light activity as 1.6 to 3 MET, moderate as 3 to 6 MET (e.g., brisk walking), and intense as 6+ MET (e.g., jogging) [3]. 

### 2.8. Secondary Outcomes

Self-reported energy and relaxation levels were measured twice-daily by ecological momentary assessment (EMA) [20] questions via the survey website link emailed at two random times during the workday (1 minute completion time). The EMA included questions about relaxation, calmness, energy, fatigue, hunger and overall well-being, on a scale of 1 to 5 where 1 indicated ‘not at all’ and 5 indicated ‘extremely’ (e.g., “How relaxed do you feel right now?”). Participants were also asked if they were standing or sitting while answering the survey, which served as another indicator of sitting *vs.* standing. 

Self-reported energy intake and nutrient intake was assessed using a web-based 24 h dietary recall (ASA-24, National Cancer Institute). The access to the survey website was sent on a randomly selected day each week and respondents were asked to complete the survey as soon as possible (20–30 min survey duration). Multiple 24 h diet recalls are deemed the best method for subjective dietary assessment [21,22]. 

Self-reported productivity was assessed using the validated “Work Productivity and Activity Impairment Questionnaire” (WPAIQ), which was emailed to the participants once a week [23]. 

### 2.9. Compensation

Participants were compensated for their time up to $150 for completion of the study and the option of keeping their SSDs at the end of the study.

### 2.10. Randomization

A member of the research team, who was not part of the enrollment or data collection process, randomly assigned each participant to receive intervention during period 1 or period 2, using a 1:1 allocation in 1 block of 35, using Microsoft Excel 2007. It was not possible to conceal allocation or blind participants or researchers given the nature of the intervention.

### 2.11. Statistical Analysis

Mixed-model repeated measures linear regression was used to analyze continuous outcomes data with SAS *(‘Proc Mixed’,* SAS 9.2, Cary, NC, USA). Statistical adjustment for order and period effects were made. Adjustment for other covariates, such as age, sex, or body mass index was not necessary because in a cross-over design, each person serves as his/her own control. No *a priori* hypotheses were made regarding effect measure modification. A type I error of α < 0.05 was accepted as statistically significant. 

## 3. Results

**Figure 1:**
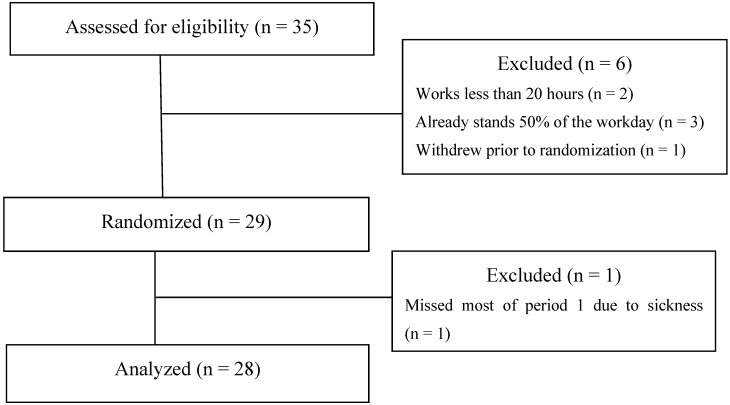
Participant flow (cross-over design). January–April, 2012, Minneapolis, MN, USA.

Figure 1 demonstrates a participant flow chart for the cross-over design. 35 participants were assessed for eligibility, six did not meet inclusion criteria; 29 were randomized. 17 were allocated to receive the intervention during period 1 and the other 12 were to receive the intervention during period 2. One participant missed most of the control period due to illness and therefore was excluded from the rest of the study, leaving 28 participants for analysis. 28 participants (19 female) took part in the study who were on average 40.4 years of age, with mean body mass index of 25.6 (SD = 4.7), and average work schedule of 36.8 (SD = 5.6) hours per week. 

**Figure 2:**
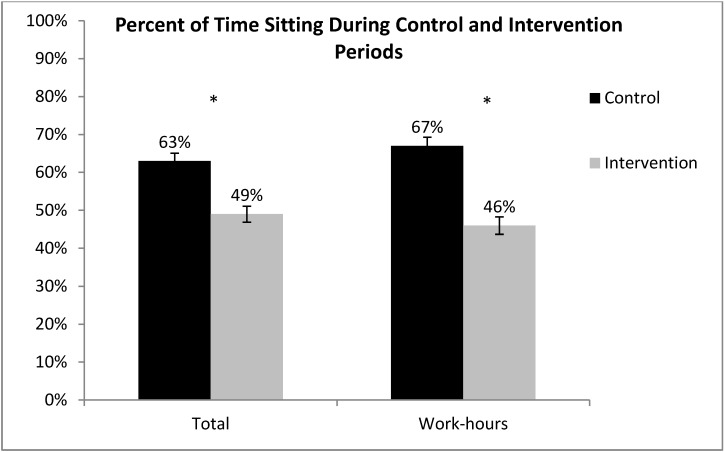
Percent of time spent sitting, during the entire day and during work-hours only, measured using the objective MSR accelerometer (*p < 0.05). January–April, 2012, Minneapolis, MN, USA.

There were 365 days of valid data (weekdays and weekends) from 28 participants using the MSR accelerometer. As shown in Figure 2, during the control period participants spent about 67% of the work-time sitting, compared to the intervention period when they sat about 46% of the work-time, a reduction of 21% in sitting time at work (95% CI of 18% to 25%). In terms of the whole day (all waking hours), participants sat about 63% of the time during the control period and 49% of the time during intervention; a 14% reduction of overall sitting time (95% CI of 11% to 17%).

There were 156 completed surveys by 26 participants for the self-reported OSPAQ. Participants reported decreasing sitting time by of 40% (95% CI: 36% to 44%) and increasing standing time by 39% (95% CI: 35% to 43%) between intervention and control periods. Walking and heavy work, which were also assessed on this survey, were not different between the two periods. 

**Figure 3:**
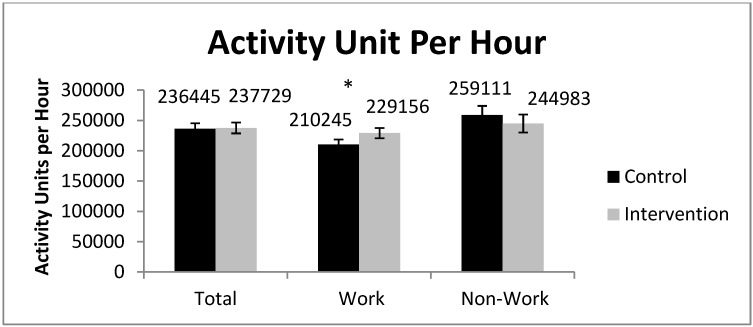
Total activity, activity during work, and activity outside of work, measured objectively by the Gruve accelerometer (*p < 0.05). January–April, 2012, Minneapolis, MN, USA.

**Figure 4:**
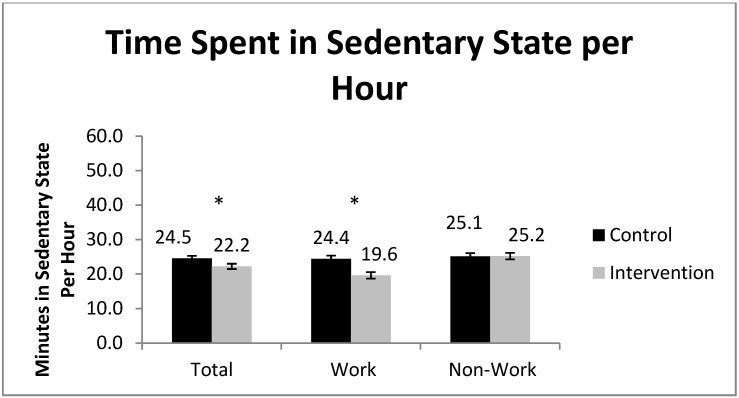
Sedentary time per hour, for total hours, work-hours only, and non-work-hours, measured objectively by the Gruve accelerometer (*p < 0.05). January–April, 2012, Minneapolis, MN, USA.

Gruve accelerometer data is based on 15,559 h (weekdays and weekends) of valid data; 7,729 h were during work-time and 7,432 h were during non-work time (evenings and weekends). AU/hr during work hours was significantly higher during the intervention compared to the control (Figure 3). However, AU/hr for total hours and non-work hours were not statistically different between CP and IP (Figure 3). Activity was further analyzed as sedentary time (Figure 4). During work hours of the control period the mean sedentary time was 24.4 min/hr, compared to 19.6 min/hr during the intervention; a difference of 4.8 min/hr (95% CI 4.14 to 5.39 min/hr). Total activity results demonstrate that during the control period participants spent 24.5 min/hr sedentary, compared to 22.2 min/hr during the intervention, a difference of 2.3 min/hr (95% CI = 1.78 to 2.86 min/hr). Activity during non-work hours was similar between the intervention and control periods.

Results of EMA are shown in Table 1, which is based on 1455 completed surveys from 28 participants. Participants reported being significantly more relaxed, calmer, more energetic, less tired, less sluggish, and felt a higher overall sense of well-being during the intervention period compared to the control. Participants also reported feeling less hungry during the intervention period, although this was not statistically significant (*p* = 0.06). During the intervention period participants reported standing 70% of the time while completing the EMA questions, compared to 2% of the time during the control period.

**Table 1:** Results of the Ecological Momentary Assessment Analysis.

	Control Period (mean ± se)	Intervention Period (mean ± se)	Treatment Effect (*p*-value)
Relaxed	3.38 ± 0.09	3.46 ± 0.09	<0.05
Calm	3.41 ± 0.09	3.52 ± 0.09	<0.01
Energetic	3.20 ± 0.10	3.28 ± 0.10	<0.05
Not Tired	3.59 ± 0.11	3.68 ± 0.11	0.05
Not Hungry	3.74 ± 0.09	3.86 ± 0.09	0.06
Not Sluggish	3.90 ± 0.12	4.02 ± 0.12	0.01
Overall Wellness	3.39 ± 0.07	3.47 ± 0.07	<0.01
Standing (proportion)	0.02 ± 0.02	0.69 ± 0.02	<0.0001

Data are mean estimates form a scale of 0 (low) to 5 (high), except for Standing, which is a proportion based on coding 0 for sitting and 1 for standing. January–April, 2012, Minneapolis, MN, USA.

There were 150 completed 24-hour dietary recalls by 26 participants. Participants reported consuming an average of 2037 (standard deviation 86.7) kcal per day during the control period and an average of 1826 (86.4) kcal during the intervention period, with a mean difference of 212 kcal (95% CI: 45 to 379 kcal; *P* = 0.01). The nutrient analyses demonstrated consistently lower intake of all macronutrients for the intervention period *vs.* control period: protein (9.0 g (95% CI: 0.6 to 17.5 g; *P* = 0.04); fat 8.8 g (95% CI: −0.6 to 18.2 g; *P* = 0.07); carbohydrate (17.2 g (95% CI: −5.1 to 39.4 g; *P* = 0.13)). 

Out of the 28 eligible participants, 26 had at least one survey completed from each period, with a total of 172 completed surveys. There was no significant difference in self-reported productivity during the intervention or control period in terms hours worked (38.3 during control *vs.* 37.1 during intervention, *p* = 0.16), hours missed due to vacation/holiday (1.52 *vs.* 1.58, *p* = 0.93), hours missed due to health reasons (2.25 *vs.* 1.24, *p* = 0.44), health impact on productivity (0.66 *vs.* 0.66, *p* = 0.99), and health impact during non-work hours (1.01 *vs.* 0.74, *p* = 0.28). 

Most participants reported experiencing increased fatigue, especially in the lower back and lower extremities, during the beginning of the intervention as they were adjusting to the SSDs and increased standing time at work. For all participants, the back fatigue/discomfort was no longer present by the second week of the intervention. The major complaint regarding the SSDs from usability perspective was the loss of work-surface compared to traditional sitting desk. Despite that, 26 out of the 28 participants at the end of the study reported willingness to continue using SSDs beyond the end of the study, thus had the desks permanently installed. 

## 4. Discussion

In this study sedentary office workers were asked to replace 50% of their sitting time during work with standing by the use of sit-stand workstations over the course of four weeks. The objective MSR accelerometer measure estimated 21% absolute decrease in sitting time, which translates into a decrease of 8 h of sitting during a 40 h work week. The estimate that included sitting time for non-work hours (evenings and weekends) suggested that the participants were not sitting more at home to compensate for the increased standing at work.

Activity during work hours was significantly higher during the intervention than during the control period as measured by the Gruve accelerometer. Participants did not compensate for higher amounts of activity during work hours by being less active during non-work hours. One reason the total activity was not significantly increased during the intervention may be that the increase in movement that comes from using sit-stand workstations is in the light activity spectrum of low MET values, and thus it would be difficult to accurately detect such differences in low intensity non-sedentary activity. The Gruve accelerometer detects walking down to a speed of 0.5 mph, however, the shift from sitting to standing at a workstation probably includes ample periods of movement that are below this threshold. Thus, even though prolonged sedentariness is being disrupted by use of SSDs, total PA changes may not be detected. The fact that prolonged sedentary time was being disrupted as a result of the present intervention is clear when the accelerometer data are broken down into the more meaningful activity spectrum which demonstrated that participants replaced about 38 min of sedentary time per day with non-sedentary time.

One of the most interesting findings from this study is that installation of SSDs—which allows people to replace sitting with standing—is effective at increasing not only standing time, but also light movement during work-hours. The findings of this study are similar to the few previous studies on this topic. One experimental study in first grade classrooms comparing SSDs with traditional desks found significantly higher caloric expenditure in the treatment group [15]. A study focused on reducing sitting time among office workers found that a number of activity promoting behaviors during work and non-work hours resulted in a 48 min decrease in sitting time during waking hours [14]. A recent SSD intervention in Australian office workers showed a decrease in sedentary time of 137 min/day at the workplace and 97 min/day overall at three month follow-up [13].

Per the EMAs, participants reported being significantly more relaxed, calm, energetic, and less tired/sluggish, and felt a higher overall sense of well-being during the intervention at work compared to the control period. However, the effect sizes were small and the interpretation of their meaning will depend on future studies. Still, one would only expect a subtle effect on the mood of a person during work due to the implementation of a sit-stand workstation. Feeling more energetic and less tired is somewhat counter-intuitive since standing may be more physically demanding than sitting. However, physiologic studies suggest a biological basis to increased feelings of energy and well-being from replacing some sitting time with standing. Standing, compared to sitting, recruits more muscle fibers and stimulates blood flow, which may help with alertness and maintaining energy levels and focus on certain work tasks and office interactions [7]. 

Based on the 24-hour dietary recalls, participants reported significantly lower caloric intake during the intervention period relative to control. This effect seemed to be consistent across the macronutrients, suggesting that if the effect is a real phenomenon, it may be explained by an appetite suppressing mechanism rather than a conscious behavior change towards healthier eating (e.g., higher fruits and vegetables consumption). Perhaps replacing sedentary time with standing and light activity suppresses hunger or buffers the desire to spontaneously eat. In fact, PA has been hypothesized to decrease appetite through neuroendocrine mechanisms, thus reducing caloric intake [24]. In support of the dietary intake effect that we observed, the hunger data from the EMA protocol suggested the participants felt less hungry during the intervention period relative to the control (*p* = 0.06). However, since the intervention could not be blinded, there is a possibility that the dietary results are due to desirability bias, an effort by the participants to please the investigators by reporting less food intake. Due to the fact that the electronic survey prompted the participants on random days, it expired if was not completed within a certain time period, and, most importantly, the study was about decreasing sedentary time and not about changing dietary intake, desirability bias seems unlikely.

Productivity is very difficult to measure in sedentary office workers. Thus, it is not surprising that no difference was found in productivity. This finding is in line with findings from other studies reporting that sit-stand or other active workstations do not increase, and do not hinder, productivity [25,26].

Participants enjoyed the flexibility to be able to sit or stand while working which was reflected by the overwhelming majority choosing to permanently keep the SSDs. The SSDs used for this study are in the $400 to $900 range, which is in the price range for high quality office chairs, and affordable relative to high quality office furniture that would include the desk and the chair. This study was done in the ‘real-world’, at the worksite and the natural workflow of the employees did not appear to be disrupted to any significant negative extent. No significant harms were reported during the intervention. The study appears to have potential for good external validity or generalizability to similar office-based sedentary workers. 

This study has several limitations. First, like most lifestyle interventions, the intervention could not be blinded. Investigators were also not blinded. However, the primary outcome was measured objectively with accelerometers, which should minimize bias. Sample size was small and intervention was short; however, use of a cross-over design significantly improved statistical power in this study. As with all cross-over intervention studies, bias can occur due to order (whether intervention was applied first or control was applied first), period (time of the year, seasonality, during when intervention or control was applied), and carry-over/contamination (carrying over of benefits or harms from one period to next) effects. The study design sought to minimize these effects through randomization of the order (intervention in 1st or 2nd period), adjustment for period and order in the statistical analysis, and implementation of a two-week ‘washout’ between periods to minimize carry-over effects. 

Future studies should include well-powered, long-term, randomized controlled trials with parallel design in various worksite settings. Future trials should further investigate the relevant secondary outcomes that were explored in the present study (e.g., psychological states, energy levels, appetite and dietary intake), should be done on populations who are most likely to benefit from this outcome (e.g., obese, inactive populations), and should examine clinically meaningful outcomes (e.g., body composition and chronic disease risk factors). 

## 5. Conclusions

This study demonstrated that sit-stand workstations can be implemented in the workplace for sedentary, computer-based, office workers. This intervention significantly reduced sitting time and increased standing and light activity during work-hours. The sit-stand workstations were well-received by the participants. Use of SSDs may be a feasible approach towards breaking up prolonged sedentary time, thus providing another approach to improving the environment and lifestyle towards the long-term goal of a healthier population. 
